# Correction: PGAM5 promotes lasting FoxO activation after developmental mitochondrial stress and extends lifespan in *Drosophila*

**DOI:** 10.7554/eLife.37316

**Published:** 2018-04-10

**Authors:** Martin Borch Jensen, Yanyan Qi, Rebeccah Riley, Liya Rabkina, Heinrich Jasper

Jensen MB, Qi Y, Reiley R, Rabkina L, Jasper H. 2017. PGAM5 promotes lasting FoxO activation after developmental mitochondrial stress and extends lifespan in *Drosophila*. *eLife* **6**:e26952. doi: 10.7554/eLife.26952.

Published 26, September 2017

In the Materials and methods section of the original article, subsection “Infection”, a sentence describing the culturing conditions for *P. entomophila* and *P. aeruginosa* listed the bacteria in reverse order. This incorrectly implied that *entomophila* was cultured at 37 degrees for 16h and *aeruginosa* at 30 degrees for 10h. The authors apologize for any inconvenience caused by this mistake.

The article has been corrected accordingly.

